# Brain computerized tomography reading in suspected acute ischemic stroke patients: what are essentials for medical students?

**DOI:** 10.1186/s12909-019-1781-x

**Published:** 2019-09-18

**Authors:** Chi-Hung Liu, Cheng-Ting Hsiao, Ting-Yu Chang, Yeu-Jhy Chang, Sheng-Han Kuo, Chun-Wei Chang, Chi-Jen Chen, Chien-Fu Chen, Po-Liang Cheng, Shy-Chyi Chin, Te-Fa Chiu, Jung-Lung Hsu, Peng-Wei Hsu, Tsong-Hai Lee, Chih-Hsiang Liao, Chun-Jen Lin, Li-Han Lin, Chen-June Seak, Pi-Shan Sung, Tao-Chieh Yang, Yi-Ming Wu

**Affiliations:** 1 Department of Neurology, Chang Gung Memorial Hospital, Linkou Medical Center, Taoyuan, Taiwan; 2 College of Medicine, Chang Gung University, Taoyuan, Taiwan; 3 Division of Medical Education, Graduate Institute of Clinical Medical Sciences, College of Medicine, Chang Gung University, Taoyuan, Taiwan; 4 Department of Emergency Medicine, Chang Gung Memorial Hospital, Chiayi, Taiwan; 5 Chang Gung Medical Education Research Centre, Taoyuan, Taiwan; 6 Department of Neurology, Columbia University, New York, USA; 7 Department of Radiology, Shuang-Ho Hospital, New Taipei City, Taiwan; 8 School of Medicine, College of Medicine, Taipei Medical University, Taipei, Taiwan; 9 Department of Neurology, Kaohsiung Medical University Hospital, Kaohsiung Medical University, Kaohsiung, Taiwan; 10 Emergency Department, Dalin Tzu Chi Hospital, Chiayi, Taiwan; 11 School of Medicine, Tzu Chi University, Hualien, Taiwan; 12 Department of Medical Imaging and Intervention, Linkou Medical Center, Chang Gung Memorial Hospital, Chang-Gung University, Taoyuan, Taiwan; 13 Department of Emergency Medicine, China Medical University Hospital, School of Medicine, China Medical University, Taichung, Taiwan; 14 Graduate Institute of Humanities in Medicine and Research Center for Brain and Consciousness, Taipei Medical University, Taipei, Taiwan; 15 Department of Neurosurgery, Chang Gung Memorial Hospital at Linkou, Chang Gung University, Taoyuan, Taiwan; 16 Department of Neurosurgery, Neurological Institute, Taichung Veterans General Hospital, Taichung, Taiwan; 17 Institute of Medicine, Chung Shan Medical University, Taichung, Taiwan; 18 Department of Neurology, Taipei Veterans General Hospital, and School of Medicine, National Yang-Ming University, Taipei, Taiwan; 19 Department of Radiology, Kaohsiung Chang Gung Memorial Hospital and Chang Gung University College of Medicine, Kaohsiung, Taiwan; 20 Department of Emergency Medicine, Linkou Medical Center, Chang Gung Memorial Hospital and College of Medicine, Chang Gung University Taoyuan, Taoyuan City, Taiwan; 21 Department of Neurology, National Cheng Kung University Hospital, College of Medicine, National Cheng Kung University, Tainan, Taiwan; 22 Department of Neurosurgery, School of Medicine, Chung Shan Medical University, Hospital, Chung Shan Medical University, Taichung, Taiwan

**Keywords:** Acute ischemic stroke, Brain CT reading, Delphi, Medical education, Medical students

## Abstract

**Background:**

Few systematic methods prioritize the image education in medical students (MS). We hope to develop a checklist of brain computerized tomography (CT) reading in patients with suspected acute ischemic stroke (AIS) for MS and primary care (PC) physicians.

**Methods:**

Our pilot group generated the items indicating specific structures or signs for the checklist of brain CT reading in suspected AIS patients for MS and PC physicians. These items were used in a modified web-based Delphi process using the online software “SurveyMonkey”. In total 15 panelists including neurologists, neurosurgeons, neuroradiologists, and emergency department physicians participated in the modified Delphi process. Each panelist was encouraged to express feedback, agreement or disagreement on the inclusion of each item using a 9-point Likert scale. Items with median scores of 7–9 were included in our final checklist.

**Results:**

Fifty-two items were initially provided for the first round of the Delphi process. Of these, 35 achieved general agreement of being an essential item for the MS and PC physicians. The other 17 of the 52 items in this round and another two added items suggested by the panelists were further rated in the next round. Finally, 38 items were included in the essential checklist items of brain CT reading in suspected AIS patients for MS and PC physicians.

**Conclusions:**

We established a reference regarding the essential items of brain CT reading in suspected AIS patients. We hope this helps to minimize malpractice and a delayed diagnosis, and to improve competency-based medical education for MS and PC physicians.

**Electronic supplementary material:**

The online version of this article (10.1186/s12909-019-1781-x) contains supplementary material, which is available to authorized users.

## Background

Stroke is one of the leading causes of death and disability worldwide. The most influential factor predicting the severity of disability and mortality in patients with acute ischemic stroke (AIS) is the time required to restore cerebral blood flow. Currently, AIS treatments including intravenous thrombolytic therapy (IVT) and intra-arterial mechanical thrombectomy (IA-MT), which have been shown to effectively improve stroke-related morbidity, disability and mortality [1]. The phrase “time is brain” is a reminder that AIS treatment should be completed promptly on arrival at the emergency department (ED) [1]. The assessment, evaluation and interpretation of the brain images of suspected AIS patients should therefore be completed in a timely, precise and comprehensive manner. In contrast to the IVTs in ED patients, the IVTs in in-hospital AIS patients are usually delayed [2]. Inadequate awareness of the urgency of AIS patients, insufficient knowledge of ordering and interpreting stroke examinations, and inadequate initiation and interpretation of brain computed tomography (CT) scan would result in a delay and quality gap of acute in-hospital stroke care [2, 3]. Therefore, dealing with AIS patients should also be an important entrustable professional activity (EPA) for primary care (PC) physicians in training.

Diagnostic studies are sub-competencies of patient care for ED physicians, and brain image reading is an important milestone in this sub-competency. Brain CT reading is as essential as physical examinations in daily healthcare practice. Although radiologists provide the official medical reports, all PC physicians still need to swiftly interpret CT findings in emergency cases in order to propose adequate treatment plans [4]. It would thus be beneficial for neurology residents, medical students (MS), and PC physicians to master these CT reading skills, including comprehensive reading, important positive and negative signs of a main diagnosis and major differential diagnoses. In patients presenting with suspected AIS, the goals of brain CT reading are not only to select the patients who are candidates for IVT and IA-MT, but also to identify the patients who have symptoms mimicking AIS [5]. Missing the subtle signs of life-threatening diseases other than AIS, such as subarachnoid hemorrhage, epidural or subdural hemorrhage, and skull fractures may also result in a wrong diagnosis, morbidity, mortality, and severe complications if receiving IVT. Moreover, doctors of different specialties may place emphasis on different factors and read in a different sequence when interpreting the same brain images [6]. The lack of standardization and of a standard reading sequence may make instructional designs for teaching and learning brain CT reading difficult [7]. Therefore, the goal of this study was to obtain a consensus on essential items and to establish a checklist for brain CT readings of patients with suspected AIS for MS and PC physicians. We applied a web-based modified Delphi method over several rounds to build consensus among a selected panel of experts.

## Methods

### The generation of a list of essential items for brain CT images of suspected AIS patients

Three attending physicians (TYC, SCC, and CJS) from the Neurology, Radiology, and ED at Chang-Gung Memorial Hospital, Linkou were enrolled in the pilot group to draft a list of essential items of brain CT images from patients with suspected AIS. All three participants had actively contributed to the treatment of AIS for more than 5 years. Their draft list was further discussed during the Delphi process. The Ethics Institutional Review Board of Chang Gung Memorial Hospital approved this study (IRB NO. 201601984B0).

### The modified web-based Delphi process

It is important to consider diversity and representativeness during the selection of panelists in the modified Delphi process [8, 9]. We selected 15 expert panelists for the modified Delphi process, including three general neurologists, three stroke neurologists, three neurosurgeons, three neuroradiologists, and three ED physicians from different medical schools and medical centres. All these panelists were experienced in brain CT reading and have actively contributed to clinical care and teaching of neurological diseases for more than 5 years. Given the busy schedule of the clinicians, getting them together at the same time would have been difficult. Therefore, we designed a web-based, anonymous, and modified Delphi process, with a dedicated URL to the free software SurveyMonkey (www.surveymonkey.com), to calculate the rating results of each item on the checklist inspired by existing literature [10, 11]. Of note, all of the chosen panelists should be familiar with a mobile device. We used personal e-mails as the main connection between the principal investigator and the panelists. First, we sent a background survey regarding their instructing experience and expertise to all of the panelists. We then started the first round of the modified Delphi process. A personalized follow-up reminder e-mail message was sent to absent panel members with a uniform resource locator link to the survey and to the webpage. The list of responders from each round was copied into new a recipient list for the subsequent rounds. All ratings, suggestions, and discussions were recorded, transcribed and anonymized.

**Fig. 1:**
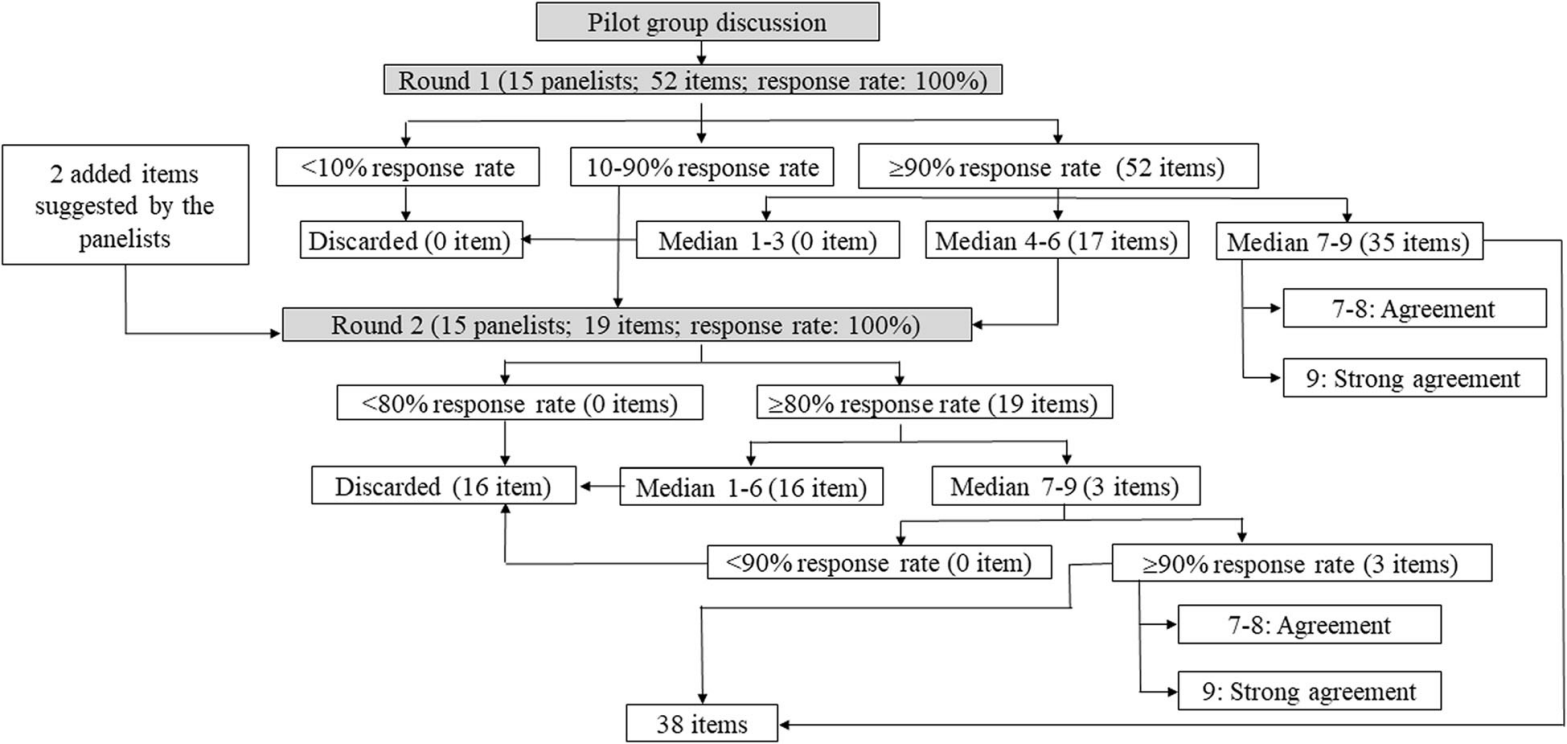
Flow chart of the pilot group and modified Delphi process. This figure shows the evolution and decision making process of all the essential items for brain CT reading in suspected acute ischemic stroke patients concluded from the pilot group and each round of the modified Delphi process. CT, computerized tomography

In the first round of questionnaires, the 15 panelists were asked to rate for whether each item should be included or excluded. The survey was conducted by mailing a questionnaire with the draft checklist to all panelists. Each panelist indicated their agreement or disagreement by using a 9-point Likert scale (1 = strongly disagree; 9 = strongly agree). The response rate indicated the proportion of the panelists who rated on each item. We discarded the items with a less than 10% response rate from the panelists. The items with response rates between 10 and 90% were discussed in the next round, while the items with a response rate over 90% were discussed in this round. Among these, the items with a median rating score of 9 were defined as “strong agreement”, those with median rating scores of 7 and 8 were defined as “agreement”, and those scored between 4 and 6 were discussed in the next round. Finally, the items with a median rating score between 1 and 3 were discarded (Fig. 1). We also asked the 15 panelists to provide feedback and suggest important positive or negative signs that were not initially included.

During the second round, we rated the items without a conclusive consensus, feedback and the items suggested by the panelists in the first round. Different to the first round, the items with a response rate of less than 80% from the panelists were discarded. Of those with a response rate over 80%, the items with a median rating score between 1 and 6 were left out regardless of their response rates; those with a median rating score between 7 and 9 were saved only if the response rate was more than 90% (Fig. 1). The items with a median rating score of 9 were defined as “strong agreement”, and those with median rating scores of 7 and 8 were defined as “agreement”.

### Statistical analysis

All statistical analyses were performed using the Statistical Package for the Social Sciences (IBM SPSS Statistics version 22.0). The Likert scale rating for each item from all of the panelists was expressed as median (quartile 1, quartile 3). Items with median scores between 7 and 9 in the first or second round were defined as being recommended brain CT reading items. In addition, we compared differences in perspective between the stroke specialists and non-stroke specialists using the Mann-Whitney U test (nonparametric data). Statistical significance was set at *p* < 0.05.

## Results

### The proposed essential brain CT items for the MS and PC physicians from the pilot group

After reviewing text books and published important image signs [12], a total of 52 essential items of brain CT reading were initially proposed from the pilot group [13–30]. All of these items were categorized into six parts, including checking the patients identification (ID), checking CT scan quality, reading bone windows, extra-axial lesions, intra-axial lesions, and identifying abnormal densities on brain CT.

### Background characteristics of the panelists

Among the 15 panelists, eight (53.3%) had more than 11 years of experience in instructing brain CT reading to students, and seven (46.7%) had less than 10 years of experience. The panelists included three (20%) stroke neurologists, three (20%) non-stroke neurologists, three neurosurgeons (20%), three neuroradiologists (20%), and three ED physicians (20%). The most common expertise of the panelists was cerebrovascular disease (53.3%), followed by vascular intervention (26.7%), spinal disease (26.7%), neuro-critical care (26.7%), medical education (26.7%), neuroimaging (20.0%), brain tumor (20.0%), and emergency medicine (20.0%). The panelists in this study therefore covered most fields of subspecialties of clinical neuroscience (Table 1).

**Table 1:** Background characteristics of the panelists

Variables	n (%)
Duration of instructing brain CT reading to students	
0–10 years	7 (46.7)
11–20 years	4 (26.7)
> 20 years	4 (26.7)
Subspecialties and academic rank of the panelists	
Neurologists	6 (40.0)
Neurosurgeons	3 (20.0)
Neuroradiologists	3 (20.0)
Emergency department physicians	3 (20.0)
Academic rank	
Professor	2 (13.3)
Assistant professor	6 (40.0)
Lecturer	7 (46.7)
Subspecialties	
General neurology	2 (13.3)
Cerebrovascular disease	8 (53.3)
Cognitive neuroscience and dementia	2 (13.3)
Epilepsy	1 (6.7)
Movement disorder	1 (6.7)
Brain tumor	3 (20.0)
Spinal disease	4 (26.7)
Emergency medicine	3 (20.0)
Neuro-critical care	4 (26.7)
Neuro-imaging	3 (20.0)
Vascular intervention	4 (26.7)
Head and neck cancer	1 (6.7)
Medical education	4 (26.7)

*CT* computerized tomography

### Results of the modified Delphi process

All of the 15 panelists completed the first and second rounds of ratings (Response rates of the both rounds: 100%; Fig. 1). All 52 of the essential items of brain CT reading were used for the first round of the modified Delphi process, of which 35 (67.3%) had agreement or strong agreement. Of these 35 items, three were strongly recommended as essential brain CT reading items for MS and PC physicians with a rating of 9, including checking the patient’s ID, identifying a mass effect, mid-line shift, or herniation, and identifying low density lesions (edematous lesions and recent infarcts). The other 17 (32.7%) of the 52 items were entered into the second round for further confirmation. The panelists also provided feedback in the first round (Additional file 1: Table S1) and suggested adding another two items to the essential list of brain CT reading in the first round (Fig. 1), which were identifying the image symmetry of the bilateral cerebral hemispheres, and reviewing the symmetry, hyperdensity, and hypodensity of the bilateral cerebellar hemispheres.

Among the 19 items discussed in the second round of the modified Delphi process, only three (15.8%) were regarded to be essential brain CT reading items for MS and PC physicians, while the other 16 items were discarded after thorough consideration. Finally, 38 items were included in the recommended essential list of brain CT reading items for MS and PC physicians (Tables 2 and 3; Additional file 1: Tables S2 and S3).

**Table 2:** Checklist of brain CT reading in patients with suspected acute ischemic stroke

1. Check patient’s ID	
2. Reading bone windows [13, 29]	
Identify skull fracture	
Identify skull destruction	
Identify skull mass lesion or osteoblastic lesion	
3. Sequential reading from extra-axial to intra-axial [16]	
4. Identify the image symmetry of the bilateral hemispheres	
Extra-axial lesions	
Epidural and subdural space [29]	
Interhemispheric fissure	
Sylvian fissure	
Ventricles	
Lateral ventricles	
Anterior and posterior horns of lateral ventricles	
Temporal horns of lateral ventricles	
Fourth ventricle	
Specific regions	
Cerebellopontine angle	
Sella lesion	
Vessels	
Hyperdense MCA sign [15]	
Hyperdense BA sign [14]	
Veins: Dense sinus signs of CVT [22]	
Orbital cavity (Ophthalmic vein enlargement, orbital mass) [17]	
Intra-axial lesion	
ACA territory	
PCA territory	
MCA territory, basal ganglia and thalamus [24]	
Borderzone areas [26]	
MCA-ACA border zone	
MCA-PCA border zone	
Temporal lobes	
Mass effect, mid-line shift, or herniation [25]	
Brain stem	
Mid-brain	
Pons	
Medulla	
Cerebellum: symmetry, hyperdensity, hypodensity of the cerebellar hemispheres.	
5. Identify abnormal densities on brain CT [27, 28]	
Identify hyper-density lesions	
Hematoma density	
Physiological calcification density	
Identify low density lesions	
Very low density (CSF and old lesions)	
Low density (edematous lesion and recent infarcts)	
Identify heterogeneous density lesions	
Hematoma with blended sign [18], whirl sign [19], spot sign [21], or black hole sign [20]	
Low density mixed with hyper-density (hemorrhagic infarct)	
Identify mass-like lesions	
ABBBC (Air-Blood-Bone-Brain-CSF) mnemonic [30]	

*ACA* anterior cerebral artery, *CSF* cerebrospinal fluid, *CT* computerized tomography, *CVT* cerebral venous thrombosis, *ID* identification, *MCA* middle cerebral artery, *PCA* posterior cerebral artery

**Table 3:** Ratings of the essential items from the modified Delphi process

	Rating scores	
9 (Strong agreement)	8 (Agreement)	7 (Agreement)
1. Check patient’s ID	1. Sequential reading from extra-axial to intra-axial	1. Identify skull fracture
2. Mass effect, mid-line shift, or herniation	2. Identify the symmetry of the bilateral hemispheres	2. Identify skull destruction
3. Low density (edematous lesion and recent infarcts)	3. Epidural and subdural space	3. Identify skull mass lesion or osteoblastic lesion
	4. Lateral ventricles	4. Interhemispheric fissure
	5. Anterior and posterior horns of lateral ventricles	5. Sylvian fissure
	6. ACA territory	6. Temporal horns of lateral ventricles
	7. PCA territory	7. Fourth ventricle
	8. MCA territory, basal ganglia and thalamus	8. Cerebellopontine angle
	9. Pons	9. Sella lesion
	10. Hematoma density	10. Hyperdense MCA sign
	11. Physiological calcification density	11. Hyperdense BA sign
	12. Very low density (CSF and old lesions)	12. Veins: Dense sinus signs of CVT
	13. Identify mass-like lesions	13. Orbital cavity (ophthalmic vein enlargement, orbital mass)
		14. MCA-ACA border zone
		15. MCA-PCA border zone
		16. Temporal lobes
		17. Mid-brain
		18. Medulla
		19. Symmetry, hyperdensity, and hypodensity of the bilateral cerebellar hemispheres.
		20. Hematoma with blended sign, whirl sign, spot sign, or black hole sign
		21. Low density mixed with hyper-density (hemorrhagic infarct)
		22. ABBBC (Air-Blood-Bone-Brain-CSF) mnemonic

*ID* identification, *MCA* middle cerebral artery, *ACA* anterior cerebral artery, *PCA* posterior cerebral artery, *CVT* cerebral venous thrombosis, *CT* computerized tomography, *CSF* cerebrospinal fluid

### Differences in viewpoints between the stroke and non-stroke specialists

Comparing the eight stroke specialists with the seven non-stroke specialists, the stroke specialists placed less emphasis on epidural and subdural spaces [stroke specialists vs. non-stroke specialists, 6.5 (6, 8) vs. 8 (7, 9), *p* = 0.03], lateral ventricles [stroke specialists vs. non-stroke specialists, 6.5 (5.3, 8) vs. 9 (8, 9), *p* < 0.03] including anterior and posterior horns and temporal horns [stroke specialists vs. non-stroke specialists, 6 (5.3, 7.8) vs. 8 (8, 9), *p* < 0.01], third ventricle [stroke specialists vs. non-stroke specialists, 5.5 (5, 6) vs. 7 (7, 8), *p* = 0.01] and fourth ventricle [stroke specialists vs. non-stroke specialists, 6 (6, 6) vs. 7 (7, 8), *p* = 0.02]. The stroke specialists also gave a lower rating for reading the medulla [stroke specialists vs. non-stroke specialists, 6.5 (5, 7) vs. 8 (8, 8), *p* = 0.03] (Table 4).

**Table 4:** Differences in the viewpoints between the stroke and non-stroke specialists in the modified Delphi process

Items of brain CT reading	Overall	Stroke specialists vs. non-stroke specialists		
		Stroke specialists	Non-stroke specialists	*p*
	*N* = 15	*N* = 8	*N* = 7	
Epidural and subdural space	8 (6,8)	6.5 (6,8)	8 (7,9)	**0.03**
Lateral ventricles	8 (6,9)	6.5 (5.3,8)	9 (8,9)	**0.03**
Anterior and posterior horns of lateral ventricles	8 (6,8)	6.5 (5.3,7.8)	8 (8,9)	**< 0.01**
Temporal horns of lateral ventricles	7 (6,8)	6 (4.3,7)	8 (8,9)	**< 0.01**
Third ventricle	6 (5,7)	5.5 (5,6)	7 (7,8)	**0.01**
Fourth ventricle	6 (6,7)	6 (6,6)	7 (7,8)	**0.02**
Corpus callosum	5 (5,7)	5 (4,5.8)	7 (5,7)	**0.03**
Medulla	7 (5,8)	6.5 (5,7)	8 (8,8)	**0.02**

Median (Q1, Q3) is used for reporting rating scores

Data were analyzed using Mann–Whitney U test. **Bold** ***p*** values are significant

## Discussion

Brain CT reading is a key process in the diagnosis and treatment of AIS. Our modified Delphi study provided a useful checklist for essential items of brain CT reading in suspected AIS patients. A standardized and sequential reading from each anatomical location may reduce errors in interpreting brain images in these patients. Although there were some studies discussing the reading patterns of neurologists, agreements between different specialties, and common errors in brain CT interpretations [28, 31, 32]. The structured checklist of brain CT reading for MS and PC physicians remain scarce. Furthermore, the common sites of residents’ misinterpretation in these studies were thoroughly discussed during our Delphi process [28]. A mobile stroke team or pre-hospital telemedicine may allow for faster image reading and treatment of AIS in the current era [33–35], however insufficient manpower with regards to stroke specialists could be a potential problem worldwide. A delay or misinterpretation of brain CT may result in a delay of door-to-needle time and decrease the potential for brain tissue salvage [36]. A previous study further demonstrated that the image-to-needle time could be a more common contributor to a delay in timely thrombolytic therapy [37]. Therefore, improving the competency of stroke image interpretation of MS and PC physicians should also play an important role in the quality of AIS care [38].

AIS is a life threatening disease, and IA-MT or earlier decompressive craniectomy has demonstrated clinical benefits in selected patients [39, 40]. Being unaware of the possible signs of large vessel occlusion (LVO) may delay the initiation of the IA-MT process [41]. The consensus of our panel suggested the importance of dense middle cerebral artery and dense basilar artery signs when reading brain CT. These signs are clues to LVO, and identifying these signs early may help prompt transferal to a stroke center or the initiation of IA-MT. Our study also suggested that the most important items when interpreting the brain CT of suspected AIS patients include low density lesions suggesting recent infarcts, a mass effect, mid-line shift, and herniation. We suggest that identifying and properly managing these life-threatening conditions in a timely manner by neurosurgeons should be a crucial EPA for all clinical physicians in training [1]. Recognizing emergency imaging findings and describing abnormalities on brain CT should also be an important milestone in the sub-competency of patient care for MS and PC physicians. Mastering the important signs of brain CT reading should be integrated into the EPA of AIS patient care. We hope our work may help to guide brain CT reading for MS and PC physicians.

Being too focused on one diagnosis can be a major source of diagnostic errors [42]. We noted that there was a discordance between the stroke and non-stroke specialists when rating the items in our study, and the stroke specialists placed less emphases on reading the medulla, ventricles, epidural and subdural spaces (Table 4). The incidence of stroke may increase after traumatic brain injury, and the risk of traumatic brain injury and associated mortality could also be increased in stroke patients [43, 44]. Of note, obstructive hydrocephalus and brain stem compression may cause mortality in space-occupying cerebellar infarcts [45]. In addition, managing hydrocephalus may be an emergency in patients with raised intracranial pressure [46]. It is essential to read the ventricles, epidural and subdural spaces carefully regardless of whether or not there are prominent signs of AIS. A structured reporting template can help to reduce errors in interpreting these findings [7]. We hope our standardized reading sequence may remind all physicians not to miss these important findings other than AIS.

There were several strengths to our modified Delphi study. First, we overcame the barriers of distance, time, and expense and achieved a high adhesion rate through this web-based method [47]. Second, we expanded the heterogeneity of our panelists. We enrolled four specialty groups (neurologists, neurosurgeons, radiologists, and ED physicians), and we expected that this could enrich the viewpoints of the panelists [48]. However, there are also several limitations to this study. First, on-line panel discussion may be associated with lower levels of interaction and engagement [49], and this could prejudice the consensus gathering process. Instead, we anonymously and individually provided the median ratings of each item and the overall comments of the panelists to reduce this confounding. Second, not all of the panelists in this study were course directors, and we only enrolled one international panelist. This may have influenced the generalizability of our results. However, we tried to include panelists from different medical centers or tertiary hospitals, and we hoped that the diversity of our panel may broaden the viewpoints in this study.

## Conclusion

We established a reference regarding the essential items of brain CT reading in suspected AIS patients for MS and PC physicians. This could be the first step towards a standardized educational materials of brain CT for MS and PC physicians by identifying the consensus among specialists. We hope this may help to minimize malpractice and a delayed diagnosis in these patients. Future studies are needed to evaluate the reliability and validity of this tool.

## Additional file

Additional file 1: Additional tables. (DOCX 29 kb)

## Data Availability

None.

## Abbreviations

ACA: Anterior cerebral artery
AIS: Acute ischemic stroke
CSF: Cerebrospinal fluid;
CT: Computerized tomography
CVT: Cerebral venous thrombosis
ED: Emergency department
EPA: Entrustable professional activity
IA-MT: Intra-arterial mechanical thrombectomy
ID: Identification
IVT: Intravenous thrombolytic therapy
LVO: Large vessel occlusion
MCA: Middle cerebral artery
MS: Medical students
PC: Primary care
PCA: Posterior cerebral artery
