# Supplementary material for: Brain computerized tomography reading in suspected acute ischemic stroke patients: what are essentials for medical students?
Source: BMC Med Educ. 2019 Sep 18;19:359. doi: 10.1186/s12909-019-1781-x (PMC6749686; doi:10.1186/s12909-019-1781-x)
Supplement: Supplementary file 1 — Additional tables. (DOCX 29 kb) [file 12909_2019_1781_MOESM1_ESM.docx]

Table S1. Important feedback of the panelists on the items in the first round

|  | Feedback |
| --- | --- |
| 1 | Identify the image symmetry of bilateral hemispheres |
| 2 | Unless you have lots of time to go through details. Identifying lens, eye ball position, and external auditory meatus are not commonly used skills for neurologists. |
| 3 | Checking the symmetry of sylvian fissure and lateral ventricles |
| 4 | Checking whether the of window levels and window widths of computerized tomography settings are suitable for brain imaging interpretations, |

Table S2. Ratings of all panelists and differences in the viewpoints between the stroke and non-stroke specialists in the first round

|  | Overall |  | Stroke specialists vs. Non-stroke specialists | | |
| --- | --- | --- | --- | --- | --- |
| Items of brain CT reading |  |  | Stroke specialists | Non-stroke  specialists | *p* |
|  | N = 15 |  | N = 8 | N = 7 |  |
| Check patient’s ID | 9(8,9) |  | 9(5.8,9) | 9(8,9) | 0.783 |
| Check orbitomeatal line | 6(3,7) |  | 4(2,7) | 7(5,7) | 0.172 |
| Identify lens and eye ball position | 5(3,7) |  | 6(3.5,7) | 4(3,5) | 0.217 |
| Identify external auditory meatus | 5(4,7) |  | 6(3.5,7) | 5(4,7) | 0.555 |
| Identify skull bone fracture (linear, depressed, basilar skull fracture) | 7(6,8) |  | 7(6,7.8) | 8(7,8) | 0.233 |
| Identify skull bone destruction (osteolytic bone lesion) | 7(6,8) |  | 7(6,8) | 7(5,7) | 0.337 |
| Identify skull bone mass lesion or osteoblastic lesion | 7(5,8) |  | 7(6,8) | 5(5,7) | 0.153 |
| Identify sinus air (frontal sinuses, sphenoid sinuses, maxillary sinuses, ethmoid air cells, and mastoid air cells) | 6(5,7) |  | 6(5,6.8) | 7(5,7) | 0.811 |
| Sequential reading from extra-axial to intra-axial (vertex to neck or neck to vertex) | 8(6,8) |  | 6(5,8) | 8(7,9) | 0.075 |
| Epidural and subdural space | 8(6,8) |  | 6.5(6,8) | 8(7,9) | **0.031** |
| Interhemispheric fissure | 7(6,8) |  | 6.5(5.3,7.8) | 7(6,9) | 0.283 |
| Sylvian fissure | 7(6,8) |  | 6.5(5.3,8) | 7(6,9) | 0.48 |
| Interpeduncular cistern | 6(5,7) |  | 6(4.3,7.8) | 6(6,7) | 0.593 |
| Lateral ventricles | 8(6,9) |  | 6.5(5.3,8) | 9(8,9) | **0.027** |
| Lateral horns of lateral ventricles | 8(6,8) |  | 6.5(5.3,7.8) | 8(8,9) | **0.009** |
| Temporal horns of lateral ventricles | 7(6,8) |  | 6(4.3,7) | 8(8,9) | **0.004** |
| Third ventricle: should be <10 mm in diameter | 6(5,7) |  | 5.5(5,6) | 7(7,8) | **0.014** |
| Fourth ventricle | 6(6,7) |  | 6(6,6) | 7(7,8) | **0.021** |
| Cerebellopontine angle (to exclude CP angle tumor, VA aneurysm) | 7(5,8) |  | 6.5(4.3,7) | 7(7,8) | 0.132 |
| Pineal region (to exclude mass lesions) | 6(5,7) |  | 5.5(4.3,6) | 7(6,7) | 0.118 |
| Sella lesion (to exclude mass lesions, pituitary apoplexy) | 7(6,8) |  | 6.5(5.3,7.8) | 7(7,8) | 0.335 |
| MCA: Hyperdense MCA sign (indirect sign of MCA large infarction) | 7(6,9) |  | 8(5.3,9) | 7(6,9) | 0.635 |
| BA: Hyperdense BA sign (indirect sign of BA occlusion) | 7(6,9) |  | 7(6.3,9) | 7(6,8) | 0.438 |
| Dense sinus signs of CVT (transverse sinus, sigmoid sinus, superior sagittal sinus) | 7(5,8) |  | 7.5(5,8) | 7(4,7) | 0.445 |
| Orbital cavity (ophthalmic vein enlargement, orbital mass) | 7(4,7) |  | 6.5(4.3,7) | 7(3,7) | 0.76 |
| Paranasal sinus | 5(4,7) |  | 5(4,7) | 7(3,7) | 0.952 |
| Mastoid and external auditory meatus | 6(3,7) |  | 5.5(4,6.8) | 7(3,7) | 0.906 |
| 10 ASPECT score locations (ganglionic and supra-ganglionic levels) | 5(3,6) |  | 5(3.3,6) | 6(3,7) | 0.77 |
| ACA territory | 8(5,8) |  | 6.5(4.3,8) | 8(6,8) | 0.336 |
| PCA territory | 8(5,8) |  | 6.5(4.3,8.8) | 8(6,8) | 0.476 |
| MCA territory, basal ganglia and thalamus (internal capsule, external capsule, insula) | 8(6,8) |  | 6.5(5.3,8) | 8(6,8) | 0.251 |
| MCA-ACA border zone | 7(4,8) |  | 6(3.3,8) | 7(6,8) | 0.557 |
| MCA-PCA border zone | 7(4,8) |  | 6.5(3.3,8) | 7(6,8) | 0.768 |
| Internal border zone | 6(3,7) |  | 4.5(3,7) | 6(4,8) | 0.447 |
| Corpus callosum | 6(4,8) |  | 5.5(3.3,8) | 6(6,7) | 0.679 |
| Temporal lobes | 7(5,8) |  | 6(4,7.8) | 8(7,8) | 0.074 |
| Mass effect, mid-line shift, or herniation | 9(8,9) |  | 9(8,9) | 9(9,9) | 0.296 |
| Mid-brain | 7(5,8) |  | 6(5,7.8) | 8(6,8) | 0.166 |
| Pons | 8(5,8) |  | 6(5,8) | 8(8,8) | 0.152 |
| Medulla | 7(5,8) |  | 6.5(5,7) | 8(8,8) | **0.021** |
| Superior cerebellar artery territory | 5(3,7) |  | 5(3.3,6.8) | 7(3,7) | 0.442 |
| Anterior inferior cerebellar artery territory | 5(3,7) |  | 5(3.3,6.8) | 7(2,7) | 0.681 |
| Posterior inferior cerebellar artery territory | 5(3,7) |  | 5(3.3,6.8) | 7(3,7) | 0.442 |
| Vermis | 6(4,7) |  | 5.5(4.3,7.8) | 7(4,7) | 0.907 |
| Hematoma density | 8(7,9) |  | 9(6.5,9) | 8(7,9) | 0.421 |
| Physiological calcification density | 8(6,9) |  | 7.5(5,8) | 8(6,9) | 0.26 |
| Very low density (CSF and old lesions) | 8(6,8) |  | 6.5(4.3,8) | 8(6,9) | 0.154 |
| Low density (edematous lesion and recent infarcts) | 9(7,9) |  | 8(5.3,9) | 9(7,9) | 0.377 |
| Hematoma with blended sign, whirl sign, spot sign, or black hole sign | 7(5,8) |  | 5(5,8) | 7(5,9) | 0.476 |
| Low density mixed with hyper-density (hemorrhagic infarct) | 7(6,9) |  | 7.5(5.3,8.8) | 7(6,9) | 0.813 |
| Identify mass like lesions | 8(6,9) |  | 7.5(6.3,8.8) | 8(6,9) | 0.548 |
| ABBBC (Air-Blood-Bone-Brain-CSF) mnemonic | 7(5,8) |  | 5.5(4.3,7.8) | 7(5,8) | 0.447 |

Median (Q1, Q3) is used for reporting rating scores. *One panelist did not complete the survey.

Data were analyzed using the Mann-Whitney U test. **Bold *p*** values are significant

**T**able S3. Ratings of all panelists and differences in the viewpoints between the stroke and non-stroke specialists in the second round

|  | Overall |  | Stroke specialists vs. Non-stroke specialists | | |
| --- | --- | --- | --- | --- | --- |
| Items of brain CT readings |  |  | Stroke specialists | Non-stroke  specialists | *p* |
|  | 15 |  | N=8 | N=7 |  |
| Check orbitomeatal line | 5(2,6) |  | 5(2.3,5.8) | 5(2,8) | 0.405 |
| Identify lens and eye ball position | 5(3,7) |  | 5(2.3,6.5) | 5(3,8) | 0.679 |
| Identify external auditory meatus | 5(5,7) |  | 5.5(5,7) | 5(2,6) | 0.513 |
| Identify sinus air | 5(4,7) |  | 5(4.3,7) | 5(2,6) | 0.635 |
| Interpeduncular cistern | 6(4,7) |  | 5.5(4.3,7) | 6(4,7) | 0.723 |
| Third ventricle: should be <10 mm in diameter | 6(5,8) |  | 6(5,7.8) | 6(5,8) | 0.81 |
| Fourth ventricle | 7(5,8) |  | 6.5(5,7.8) | 8(5,8) | 0.636 |
| Pineal region | 5(4,6) |  | 5(4.3,6) | 5(4,6) | 0.859 |
| Paranasal sinus | 5(4,6) |  | 5.5(4.3,6.8) | 5(2,5) | 0.186 |
| Mastoid and external auditory meatus | 5(5,6) |  | 6(5,6) | 5(3,6) | 0.144 |
| 10 ASPECT score locations | 4(3,5) |  | 3.5(3,5) | 4(2,7) | 0.86 |
| Internal border zone | 4(3,5) |  | 4(3.3,4.8) | 5(3,6) | 0.262 |
| Corpus callosum | 5(5,7) |  | 5(4,5.8) | 7(5,7) | **0.032** |
| Superior cerebellar artery territory | 5(4,6) |  | 5(4.3,5) | 5(3,7) | 0.675 |
| Anterior inferior cerebellar artery territory | 5(4,6) |  | 5(4,5) | 5(3,7) | 0.594 |
| Posterior inferior cerebellar artery territory | 5(4,6) |  | 4.5(4,5) | 5(3,7) | 0.314 |
| Vermis | 5(5,7) |  | 5(4.3,5.8) | 7(5,8) | 0.119 |
| The learners should identify the image symmetry of the bilateral hemispheres | 8(7,9) |  | 8(7,9) | 8(6,9) | 0.591 |
| Cerebellum: the learners should grossly review the symmetry, hyperdensity, and hypodensity of the bilateral cerebellar hemispheres | 7(6,8) |  | 7(6,7) | 7(6,8) | 0.72 |

Median (Q1, Q3) is used for reporting rating scores. *One panelist did not complete the survey.

Data were analyzed using the Mann-Whitney U test. **Bold *p*** values are significant
